# The first online conference for breast cancer survivors—SURVIVA 2018: an innovative information tool

**DOI:** 10.1007/s00520-019-04810-4

**Published:** 2019-04-17

**Authors:** Fadime Cenik, Michaela Steinhart, Mohammad Keilani, Richard Crevenna

**Affiliations:** 1 Department of Physical Medicine, Rehabilitation and Occupational Medicine, Medical University of Vienna, Waehringer Guertel 18-20, A-1090 Vienna, Austria; 2 AINA GmbH, A-2392 Sulz im Wienerwald, Austria

**Keywords:** Breast cancer survivor, Online conference, Expert, Information, Support

## Abstract

**Purpose:**

The implementation of a new online conference tool, with the goal of providing competent answers, information, and support from experts in their fields, about diagnosis, treatment, and rehabilitation in breast cancer patients.

**Methods:**

The implementation process and data of the first online conference are described.

**Results:**

Following the idea and initiative of a breast cancer survivor, and under the umbrella of a leading oncologist in breast cancer treatment, and with the cooperation of further leading experts in the fields, plus their therapeutic teams, the new online conference SURVIVA 2018 was implemented as an innovative platform—free of charge, online, and with easy and anonymous access—to provide breast cancer survivors with in-depth information and help from the leading Austrian experts in their fields. This first online conference for German-speaking breast cancer survivors is an innovative and modern concept, which seems to have been very well accepted.

**Conclusion:**

This concept could also be of interest to survivors of other cancer entities.

Modern cancer treatment leads to improved survival rates and better quality of life, but this is accompanied by many questions concerning diagnosis, treatment, rehabilitation, and lifestyle factors, for which cancer survivors have a great need for relevant and competent answers [1–6]. Breast cancer patients, in particular, are able to benefit from early diagnosis, modern treatment, and from lifestyle interventions such as exercise, as well as from cancer rehabilitation [1–3]. To the best of our knowledge, the “First Online Conference for Breast Cancer Survivors – SURVIVA 2018” was the first online conference in the German language. SURVIVA was aimed at establishing a new online conference tool, with the goal to provide competent answers, information, and support from experts in their fields, about diagnosis, treatment, and rehabilitation in breast cancer patients [7].

Following the idea and initiative of a breast cancer survivor (Magister Michaela Steinhart), and under the umbrella of a leading oncologist in breast cancer treatment (Professor Guenther Steger), and with the cooperation of further leading experts in their fields (radiology: Thomas Helbich; surgery: Michael Gnant; gynecology: Christian Singer; radiation therapy: Joachim Widder; oncology/nutrition: Irene Kührer; cancer rehabilitation: Marco Hassler; and side-effect management and cancer rehabilitation: Richard Crevenna), plus their therapeutic teams, the new online conference SURVIVA 2018 was implemented as an innovative platform—free of charge, online, and with easy access—to provide breast cancer survivors with in-depth information and help from the leading Austrian experts in their fields [7]. The primary target group was breast cancer survivors, 3 years after treatment.

The initiative started with a formal written concept. Then, after inviting leading experts to participate, the program was established and the promotion of the conference started. At this stage, several technical issues had to be addressed to allow the implementation of this new and interactive online conference tool. The project has been very time-efficient, with the following milestones: the initial idea in January 2018 was followed in February 2018 by the invitation to experts to feature in online videos about different topics of their expertise.

All the technical issues and problems had been solved by April 2018. During this time, the videos were produced about topics of interest for breast cancer patients, including diagnosis, treatment, rehabilitation, and different lifestyle factors (nutrition, exercise, and the management of stress and fear, i.e., psycho-oncology). For the field of physical medicine and rehabilitation, for example, low-budget videos were produced using a smartphone of discussions with experts on the benefits of exercise for quality of life, participation, and even for survival, and also about the possible risks and contraindications [3, 6–10]. Furthermore, different films were produced with practical advice on how to exercise to improve endurance capacity, muscular strength, flexibility, and sensorimotor functions—for every day of the conference—so that the breast cancer survivors were able to educate themselves about exercise and to actively participate in real time at home. Then, on 22 May 2018, SURVIVA 2018 started with the following mottos [7]:

“A six-day conference with a new topic every day.”

“Attendance at home – easy access by online registration – all presentations free of charge.”

“Experts in their fields answer the questions (which had been sent before the production of the videos to the organizers of the conference) of breast cancer survivors.”

The participating patients were interested in cancer-related topics such as the effects and side effects of examinations and surgery, radiation therapy and chemotherapy, physical medicine, return to work, supportive care, and the effects, contraindications, advantages, and disadvantages of exercise. These topics were discussed and streamed each day in the morning session. The videos about different activities, with the invitation to participate (e.g., for improvement of flexibility, sensorimotor function, endurance, muscle strength, lymphedema, and mindfulness skills), were streamed in the afternoon session.

The practical experience confirmed the feasibility and acceptance of this pioneering project.

A total of 653 people attended this first online conference in the German language for breast cancer survivors.

Of this total, about 60% were from Austria (about 8,000,000 inhabitants) and 40% were from Germany (about 80,000,000 inhabitants). Most of the participants were in the age range of 45–55 years. There were 3768 video calls, one-third of which were live, plus the recorded videos.

The calculated expectation for the next online conference SURVIVA 2019 indicates that there is an expected potential of about 10,000 people, with an expected attendance of 2,000 participants. Seventy percent of these participants are expected to come from Germany and 30% from Austria. The number of approximately 10,000 represents about 8.3% of the primary target group, namely, breast cancer survivors, 3 years after treatment.

This first online conference for German-speaking breast cancer survivors is an innovative and modern concept, which seems to have been very well accepted, to provide information and help from leading experts by easy and anonymous access, with no charge to the participant. In our opinion, this innovative concept should be expanded (e.g., produced and organized in different languages and for different sociocultural populations) to reach and support as many breast cancer survivors as possible. Furthermore, such a concept could also be of interest for survivors of other cancer entities.
